# Hospital Festivals and Meetings

**Published:** 1894-05-19

**Authors:** 


### HOSPITAL FESTIVALS AND MEETINGS.

### ROYAL SEA-BATHING INFIRMARY, MARGATE.

In aid of the above institution a well-attended meeting was held on May 9th, by permission of the Duke of Westminster. The chair was taken by the Earl of Cranbrook (President of the hospital), and among those present were Earl Stanhope, Sir William Broadbent, Mr. M. Biddulph, M.P. (Treasurer of the institution), the Rev. Prebendary Whittington and the Presidents of the Royal College of Physicians and the Royal College of Surgeons; while letters of regret for unavoidable absence were received from the Duke of Cambridge, Mr. Locke, and the Dean of Westminster, who promised an offertory for the charity. The Chairman, in his opening address, alluded to the purpose for which the meeting was convened—to impress upon the friends of the institution the need of raising funds for extending the work. He adduced the testimony of medical men—and especially of Sir Andrew Clark and Sir Erasmus Wilson, the benefactors of the institution—as to the need for the existence of such an infirmary in order to mitigate and cure a disease fostered by the existing conditions of modern life. The first resolution—deploring the closing of 140 out of the 220 beds, and pledging the meeting to endeavour to raise the necessary funds—was moved by Lord Stanhope, who suggested that rather than leave too many beds unoccupied it would be better to receive paying patients in the, at present, empty part of the hospital at a payment of from 15s. to £1 a week. The motion was seconded by Mr. Jonathan Hutchinson, President of the Royal College of Surgeons, who said that tuberculous disease, being ever with us, did not attract so much attention as cholera and other diseases of an epidemic nature. It had been proved that the air of Margate was beneficial to those suffering from tuberculosis; and he thought the merits of the institution only wanted to be brought before the public in order to be recognized. Mr. Knight Treves, who followed, gave the number of patients received since the year 1796, at 48,000; these figures should not, he said, be compared with those of a general hospital, since the shortest period for which patients were received was two months, and they often remained for six or even twelve. The second resolution, that the public should be invited to contribute to the funds of the institution so that the full number of beds could be utilised, was moved by Colonel Haygarth, who said that it would be a disgrace to London, from which so large a proportion of the patients came, were sufficient funds not raised there for the work. The President of the Royal College of Physicians, in seconding the resolution, said he had been to Margate the week before to see the institution, and had been much struck by its favourable position. It was not only the air and sunshine, but the seaweed of which there was so great a quantity close to the building that helped to make Margate so health-giving to the patients. He had also been struck by the "beautiful" manner in which the scrofulous scars of the inmates of the infirmary had, in many cases, been healed. Among the other speakers were Sir William Broadbent, who said that they must remember that it was skilful surgery as well as the advantages of the locality which operated in the cures, and Sir Richard Wyatt, who urged that the friends of the institution should beg for it from their friends. The meeting came to an end with the passing of votes of thanks to the Duke of Westminster and the Chairman.

